# Mammalian and avian species quantification in homogenized foods: real time PCR and digital PCR as tools for label compliance controls

**DOI:** 10.1038/s41598-024-61009-2

**Published:** 2024-05-09

**Authors:** Bertasi Barbara, Tilola Michela, Mangeri Lucia, Benevenia Roberto, Cappa Veronica, Scaburri Alessandra, Scaramagli Sonia, Bergami Raffaella, Enrico Pavoni, Losio Marina Nadia, Peletto Simone

**Affiliations:** 1 Istituto Zooprofilattico Sperimentale della Lombardia ed Emilia Romagna, Via A. Bianchi 9, 15124 Brescia, Italy; 2 COOP ITALIA, Via del Lavoro 6/8, 40033 Casalecchio di Reno, Bologna, Italy; 3 Istituto Zooprofilattico Sperimentale del Piemonte, Liguria e Valle D’Aosta, Via Bologna 148, 10154 Turin, Italy

**Keywords:** Biotechnology, Molecular biology

## Abstract

Currently food fraud and authenticity of products composition are topics of great concern; ingredients quantification could allow to identify small amounts of contaminats or voluntary addition of improper components. Many molecular methods are available for species identification in foodstuffs but, for a better application, they should not be affected by the interference of other ingredients. The main purpose of this work was to verify the Real Time PCR and the Digital PCR (dPCR) quantification performances on baby food samples, specifically selected for their high miscibility to limit variability; chicken was selected as target to verify the performance of quantification of methods after having spiked the same quantity in different baby foods. The other aims were: (1) to verify a constant genome copies ratio existence between mammalian and avian species (2) to verify the dPCR performance, set up on housekeeping, to quantify mammalian and avian species in commercial products. Digital PCR showed fewer differences respect to Real Time PCR, at the same 15% w/w chicken spiking level. Despite the constant difference between mammalian and avian genome copies, in samples with the same spiking weight, the confidence intervals increasing towards the extreme values, made impossible to use genome copies ratio as a sort of correction factor between species. Finally, the dPCR system using the myostatin housekeeping gene to determine the chicken content seemed reliable to verify the labelling compliance in meat-based commercial products.

## Introduction

Species identification in complex food products is a topic of great concern in the field of food fraud and authenticity. European Regulation 1169/2011 defines that all ingredients have to be listed in labels, including low-quantity elements (below 2% of weight). At the moment, the legislation lacks the definition of cut-offs to distinguish contaminations from voluntary addition of undeclared ingredients; since 2013, a pragmatic limit of 1% w/w is being in general applying for all official controls in meat-based products after a huge European issue of meat-based products contamination by horse meat (EU recommendations 2013/99/EU). The economically motivated adulteration (EMA) is a fairly common practice to achieve illegally an economic advantage. For instance, chicken, that is less expensive, is added to other meat products such as pork or beef; otherwise, sheep or goat dairy products can be mixed with bovine milk during their working^1^. Trace contamination instead happen when production lines in the same plant are not properly separated, or when inefficient cleaning procedures are used. It is very difficult to establish a cut-off value that can define a “trace quantity”, because it can depend on different factors, like type of production, cleaning procedures, work surfaces, or production batch quantities. Therefore, to address the presence of a contaminant as accidental or suspect fraudulent addition it is essential to detect the amount of the contaminant by means of very efficient quantification methods. Among molecular applications developed for fraud identification there are PCRs methods used to detect target species in food^2–5^; many Real Time PCRs based on standard curve implementation and other molecular biology systems have been developed^6–10,12^. Reference materials standard curves can be created by different target food species mixed in various percentages, or by purified DNA mixtures. Currently, few reagent producers provide meat reference materials, anyway not for all the species of interest; so, most materials must be produced in house. When the standard meat sample is represented by a single target, it is possible to quantify its genome by spectrophotometer or fluorimeter, and create a correlation curve fitting ng/µL DNA with Ct values. Conversion curves can also be created to obtain target samples percentage by weight. Many Real Time PCR methods were developed to detect mitochondrial DNA, but such target is not ideal for quantification, due to the presence of numerous mitochondria in cells, which varies depending on tissues and species^13^. When sequences are available, single-copy DNA have to be selected as target for quantification^8^. The set-up of a quantification method is furthermore worsened by the presence of inhibitors that can interfere with the target in food matrices; moreover, quantification differences can be due to a target inhomogeneous distribution in the same sample. Digital PCR is a technique based on DNA partitioning in droplets or microwells, where specific targets are amplified on a thermocycler and detected by fluorescent signal. Partitioning follows the limiting dilution principle, and DNA target absolute quantification is performed by Poisson algorithms application^14–16^. Different studies^13,15,17,18^ demonstrated that Digital PCR systems are less sensitive to inhibitors, suggesting to be a highly suitable approach for species quantification in food compared to Real Time PCR. Furthermore, in Real Time PCR, the obtained result is largely dependent on the composition of the standard used, while digital PCR could be applied without a standard curve construction. However, also for digital PCR it is possible to use different meat weights of a selected target to create linear curves to determine the correlation between target quantity and nucleic acid content, as well as between DNA quantity and DNA copy number^19^; the combination of them allows to establish the relationship between weight and copy number. In the described conditions, dPCR was very efficient to obtain weight values starting from known DNA quantity, but it was exclusively applied on minced meat constituted of known quantity of two species.

In fact, the first aim of this study was to verify if the weight percentage associated with a defined target (chicken DNA) measured by Real Time PCR and Digital PCR, maintained the same values in matrices of different composition. The target selected for both amplifications was the *transforming growth factor beta-3 (TGFB3)* gene of chicken (*Gallus gallus)*; to avoid lack of repeatability due to target inhomogeneous distribution, homogenized baby food was selected to be spiked as being a highly miscible matrix with different ingredients, containing both animal and vegetal DNA. Data obtained by digital PCR were compared with Real Time PCR results on the same samples, to verify the impact of inhibitors.

Moreover, to develop a suitable method for species quantification in food products, it is also important to evaluate the differences, especially by digital PCR (less sensible to inhibitors), of the genome copies values obtained from different species tested at the same weight; this is fundamental to correctly calculate the weight percentage of different targets in multi ingredients samples, because, if there are differences in the number of genome copies among the various species, weight percentage cannot be inferred directly. Consequently, the second aim was to evaluate the correlation among dPCR data obtained from different species.

The third aim was to quantify the DNA of each species present in field samples purchased with multispecies ingredients. To do this, the housekeeping Myostatin gene (common to all vertebrate animals) was quantified as reference DNA in each sample, and its concentration was compared to those of the other species-specific DNAs of the animal ingredients declared on the label.

## Results

### Real time PCR assay

Means and standard deviations from raw Ct values, obtained by Real Time PCR on chicken target (15% and 1.5%) in the different spiked baby foods, highlighted an actual difference of 2.9 Cts between total mean values (raw data, mean and standard deviation are described in supplementary Tables S1, S2 and S3).

Real Time PCR method was able to detect Ct differences between spiked samples (even if the 3.3 Ct distance, associated with logarithmic differences, in case of perfect standard slope, wasn’t respected). To evaluate the differences among samples spiked at 1.5% and 15%, data were obtained from four different amplification runs with the two levels of concentrations.

After normality check, applied on each run Ct data (shapiro wilk test p > 0.05) and after variance homogeneity evaluation (Bartlett test, p < 0.05), Kruskal Wallis multiple comparison test and post hoc Dunn test were applied. Kruskal Wallis test produced a p-value < 0.05, confirming that a statistically significant difference among groups existed; Dunn test showed that Ct from 15% spiked samples differed from those spiked at 1.5% (p < 0.05—Table 1).

**Table 1:** Dunn comparisons between Ct raw data obtained from the Real Time PCR runs (samples spiked at 15% and at 1.5% weight percentage, three replicates for each run).

	z value	P value
Run 1–Run 2	1.8250732	0.082
Run 1–Run 3	2.4513548	0.02
Run 2–Run 3	0.6262816	0.5
Run 1–Run 4	− 3.8589069	2.3 × 10^–4^
Run 2–Run 4	− 5.6839800	3.9 × 10^–8^
Run 3–Run 4	− 6.3102616	1.7 × 10^–9^

For the following comparison among different foods, 15% spiked samples were selected to better verify quantification respect to possible matrix interference.

Standard curves raw data (chicken muscle and baby food with 30% of chicken) in different runs are available in Supplementary materials (Table S3).

**Figure 1:**
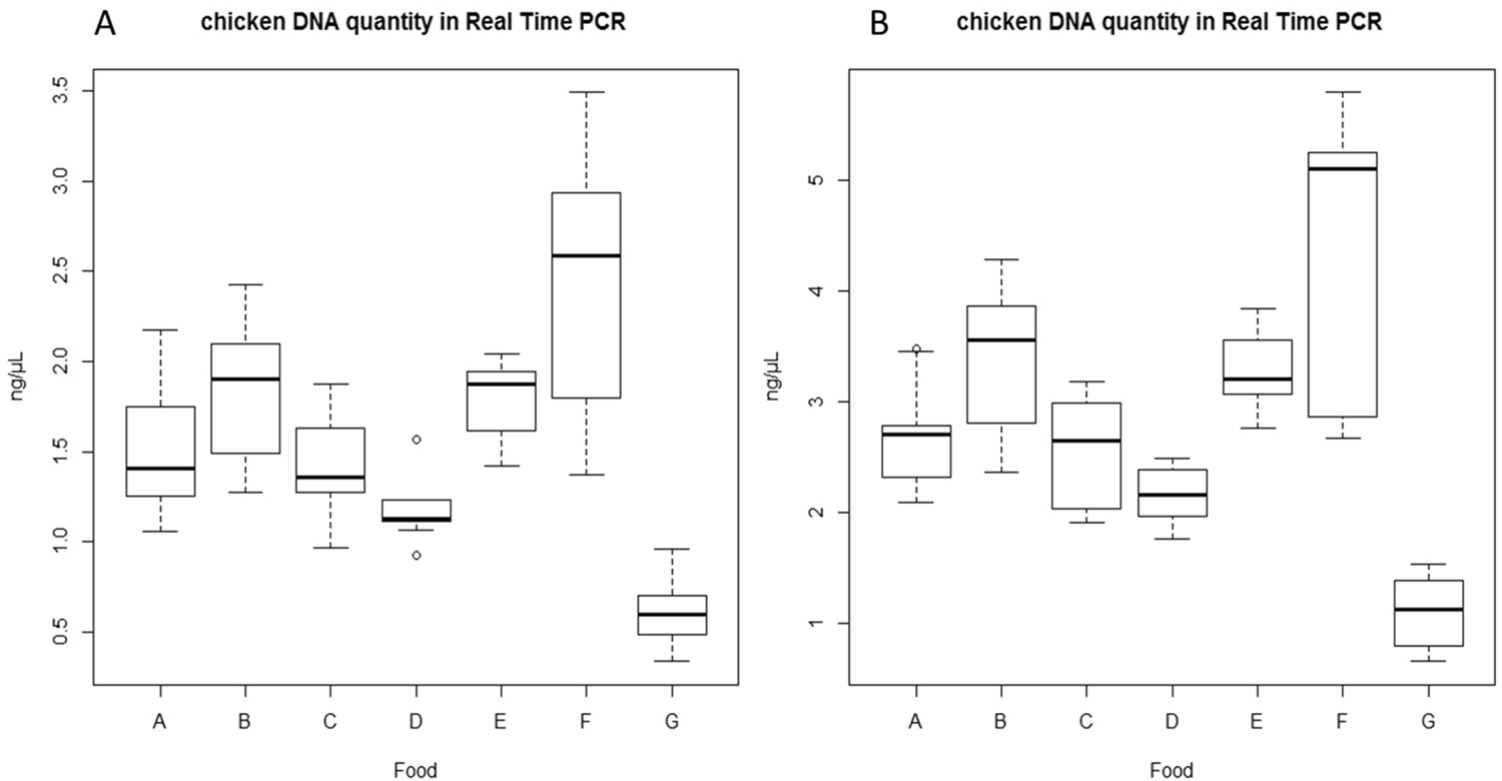
Boxplots of chicken DNA concentration in food samples spiked at 15% chicken (three runs with three replicates for each food were analysed); (1**A**) data obtained *vs* 100% chicken muscle standard curve (1**B**) data obtained *vs* 30% chicken baby food standard curve. Legend: veal meat baby food (**A**), soft cheese (**B**), organic soy mayonnaise (**C**), mixed vegetables baby food (**D**), mixed fruits baby food (**E**), Yogurt (**F**), Beef (**G**). Y axis: DNA concentration.

Nanograms data obtained from runs in Real Time PCR from different food samples were compared (Table S4). Difference among some mean values are present; boxplots show that some group could appear statistically different, in details beef baby food (G). For each food type, no difference was observed between DNA concentration values of food samples obtained from the two different standard curves used (Fig. 1).

### Real time PCR assay and digital PCR comparison

**Figure 2:**
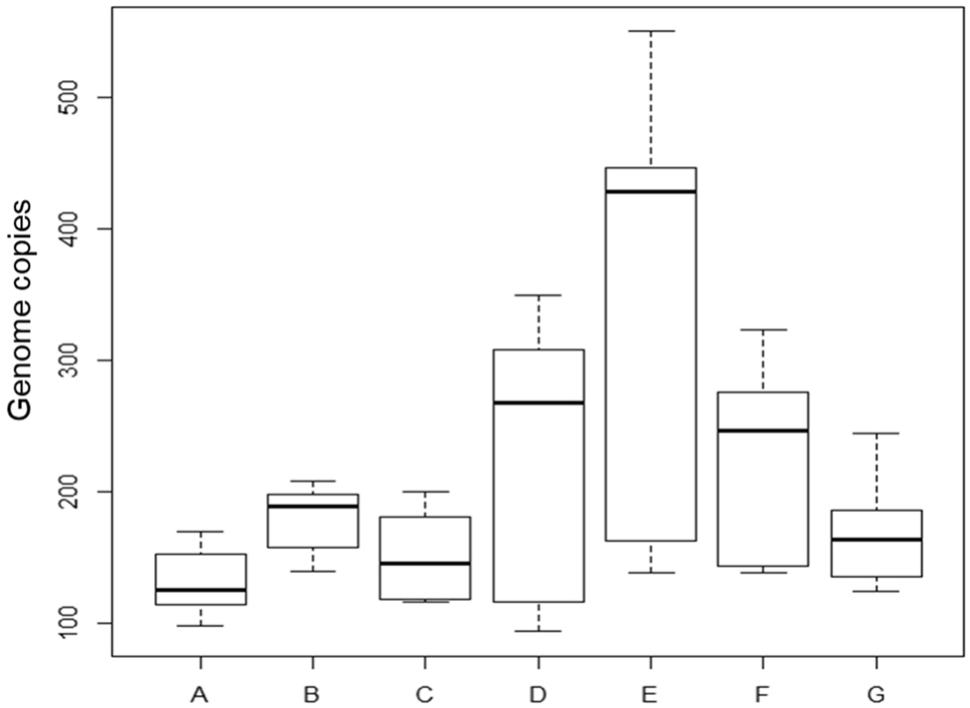
Boxplots of genome copies data obtained by dPCR from different food samples spiked at 15% (three replicates for three runs were analysed). Legend: veal meat baby food (**A**), soft cheese (**B**), organic soy mayonnaise (**C**), mixed vegetables baby food (**D**), mixed fruits baby food (**E**), Yogurt (**F**), Beef (**G**). Y axis: genome copies.

In Fig. 2 boxplots are made by absolute quantification of chicken target by digital PCR. Genome copies values were considered only if obtained by runs with valid partitions. For digital PCR chip evaluation, a 0,5 of Quality value was selected; the number of valid partitions respect to wells filled of products 15% spiked are reported in Table S6.

(Raw data are supplied in supplementary Table S5). Boxplot comparison showed that Digital PCR data were characterized by less variability respect to Real Time PCR data.

Statistic was applied to verify what was suggested by boxplot comparison.

After normality (Shapiro test p > 0.05) and variance homogeneity (Bartlett test p < 0.05) evaluation of different groups of data, Kruskal Wallis test (p < 0.05) and Dunn post hoc test was applied for intra Real Time PCR and Digital PCR comparisons; this approach was applied to verify significative differences among products spiked with the same chicken level. In Table 2 the results obtained from post-hoc test are listed.

**Table 2:** p values related to multiple comparisons (Dunn test) among raw data obtained from different type of food, with real time PCR and digital PCR (for each food three replicates in three runs for real time PCR and dPCR were analysed).

	E vs G	E vs B	G vs B	E vs A	G vs A	B vs A	E vs C
Real-time standard 1	**P < 0.0001**	P = 0.95	**P < 0.0001**	P = 0.918	**P = 0.012**	P = 0.17	P = 0.15
Real-time standard 2	**P < 0.0001**	P = 0.98	**P < 0.0001**	P = 0.17	**P = 0.009**	P = 0.17	P = 0.13
Digital PCR	P = 0.10	P = 0.25	P = 0.58	**P = 0.004**	P = 0.31	P = 0.12	**P = 0.035**

	G vs C	B vs C	A vs C	E vs D	G vs D	B vs D	A vs D
Real-time standard 1	**P = 0.015**	P = 0.14	P = 0.87	**P = 0.012**	P = 0.17	**P = 0.012**	P = 0.22
Real-time standard 2	**P = 0.015**	P = 0.13	P = 0.88	**P = 0.009**	P = 0.17	**P = 0.010**	P = 0.2
Digital PCR	P = 0.6	P = 0.32	P = 0.53	P = 0.16	P = 0.72	P = 0.75	P = 0.17

	C vs D	E vs F	G vs F	B vs F	A vs F	C vs F	D vs F
Real-time standard 1	P = 0.3	P = 0.4	**P < 0.0001**	P = 0.4	**P = 0.031**	**P = 0.018**	**P = 0.0007**
Real-time standard 2	P = 0.27	P = 0.44	**P < 0.0001**	P = 0.43	**P = 0.027**	**P = 0.017**	**P = 0.0005**
Digital PCR	P = 0.51	P = 0.49	P = 0.31	P = 0.59	**P = 0.033**	P = 0.16	P = 0.48

Significant values are given in bold.

Twenty-one multiple comparisons between foods were performed by Dunn test. In Real Time PCR there was a perfect correspondence among the p values obtained using the two different standards (for each comparison). The number of comparisons among products with p values < 0.05 was higher in Real Time PCR than in dPCR data (48% respect to 14.3%). Data obtained from fruit presented a higher variability respect to other food groups; when baby food fruit data were not considered, the percentages of p values < 0.05 were respectively 53% in Real Time PCR and 7% in dPCR.

### Digital PCR quantification of mammalian and avian species grouped

For chip evaluation a 0.5 of Quality value was selected; the number of valid partitions respect to wells filled are reported in Table S8.

**Figure 3:**
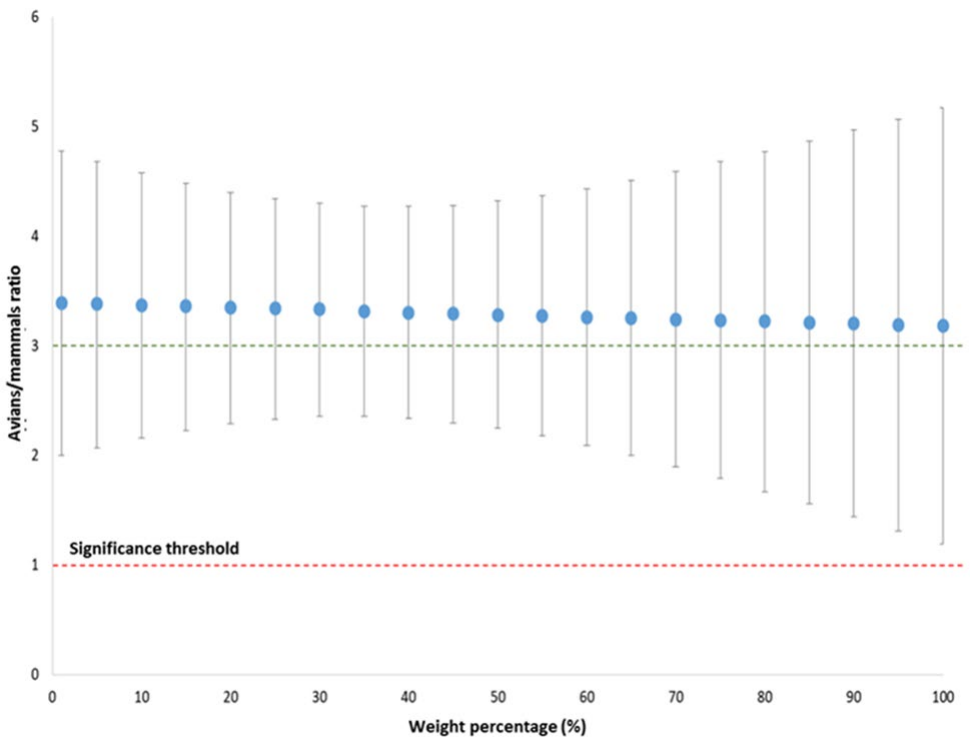
Estimation of the correction factor for species concentration (from 1 to 100%); graphic representation of avian/mammalian ratio and confidence intervals.

Table 3 shows the average number of genomic copies among weight percentage and species ratio (raw data are available in supplementary Table S7). Figure 3 shows the negative binomial distribution to verify the possibility to estimate a correction factor for avian-mammalian species concentration.

**Table 3:** Negative binomial distribution estimation of the correction factor for avian-mammalian species concentration; data (genome copies and ratios between species) obtained from dPCR in muscle samples at 100, 50, 25, 10 and 1 weight percentage (four replicates for each mammalian and avian species; four species are analysed for each group).

Weight percentage (%)	Ratio avian/mammals	95% CI
Total	3.31	[2.3;4.3]
1	3.39	[2.0;4.8]
5	3.38	[2.1;4.7]
10	3.37	[2.2;4.6]
15	3.36	[2.2;4.5]
20	3.35	[2.3;4.4]
25	3.34	[2.3;4.3]
30	3.33	[2.4;4.3]
35	3.31	[2.4;4.3]
40	3.3	[2.3;4.3]
45	3.29	[2.3;4.3]
50	3.28	[2.3;4.3]
55	3.27	[2.2;4.4]
60	3.26	[2.1;4.4]
65	3.25	[2.0;4.5]
70	3.24	[1.9;4.6]
75	3.23	[1.8;4.7]
80	3.22	[1.7;4.8]
85	3.21	[1.6;4.9]
90	3.2	[1.4;5.0]
95	3.19	[1.3;5.1]
100	3.18	[1.2;5.2]

Other percentage values were simulated.

Data suggested that avians on average had 3.31 times more genetic copies than mammals (95% CI [2.36; 4.27], p < 0.0001).

### Field samples analysis based on myostatin gene

For chip evaluation a 0.5 of Quality value was selected; the number of valid partitions respect to wells filled are reported in Table S9.

Table 4 shows the results obtained on commercial foods with the myostatin approach: percentages obtained from genome copies ratio are very similar to label declaration, for all types of products tested (raw data are presented in Supplementary Table S10).

**Table 4:** Genome copies values of species targets and myostatin (means and standard deviations of three replicates); percentages of targets respect to myostatin (representing whole meat content).

Food	Label declaration		Genome copies, means ± standard deviation	Genome copies myostatin	Target/myostatin ratio (%)
	%	species			
Hamburger	78	Bovine	21,587 ± 1905	29,294 ± 1697	73.7
	8	Swine	1886 ± 50		6.4
Meatballs	60	Chicken and turkey	182 ± 29 and 28,977 ± 7699	36,328 ± 4851	0.5 and 79.8
	19	Swine	3031 ± 68		8.3
Wurstel	46	Chicken	33,043 ± 931	61,516 ± 10,723	53.7
	38	Turkey	14,836 ± 2566		24.1

## Discussion

The application of species quantification systems is possible if they are suitable to detect the desired target into multi-ingredient foods; anti froud competent authorities need to verify the labels compliance by asking to detect undeclared species in multi-ingredients food. In this case, molecular biology is appropriate in terms of analytical sensitivity and speed, but different situations related to samples homogeneity and composition can interfere with an accurate quantification of target species. Interference can be more evident in some methods then in others: this is the case of Real Time PCR respect to dPCR. Assuming that the variability could be considered negligible in homogenized baby foods, data obtained in this work suggested that Real Time PCR was more influenced by food composition than dPCR in spiked samples. Multiple comparisons showed that the same target concentration (15% chicken) was differently quantified in the considered products by Real Time PCR: 10 of the 21 comparisons among samples were statistically significant, while only 3 comparisons in dPCR were confirmed statistically different. This evaluation was performed on samples spiked at 15% chicken, a level of contamination deliberately chosen to be high to better verify the matrix interferences.

This choice could make it possible to hypothesize that at target concentrations higher than 15% (e. g. in field samples), the variability in the detection could decrease, both for the greater probability to detect target and both for a lower effect of inhibitors.

In any case, in the context of meat fraud, the concentration selected for this work can be considered as compatible with reality.

In the study of the effect of inhibitors on different matrices by dPCR, the comparison quantification of target was different in foods containing only meat respect to products containing fruits and vegetables. This probably was due to a sort of interference by specific vegetable molecules, already known as inhibitors for Real Time PCR. Since different strategies are used to apply extraction from vegetables and fruits; a modification of the extraction system could be improved for those products that contain a higher percentage of vegetables than meat, for example by using not only appropriate lysis solutions, but also buffer and washing solutions that limit inhinibitors impact. Considering the type of control required from authorities to avoid fraud, in which sometimes quantification is required, and the type of target products, Digital PCR seems to be potentially more reliable than Real Time PCR in the quantification of species in products with multiple ingredients. In addition to the complications inherent in the type of determinations carried out, it is necessary to underline that the Digital PCR quantification is carried out using well-defined conditions of instrumental use, which determine the acceptability of the reaction, such as quantities of DNA compatible with the use of the chips, carrying out of appropriate dilutions and consequently the use of multiplication factors that allow the final result to be obtained. These complications, as is logical, must be kept in mind when applying different instrumental measurement systems.

Regarding standard selection, in the present case Real Time PCR different standards behave identically in all comparisons; in our case standards are mainly composed by meat (muscle and homogenized product with 30% of meat). However, an important difference between Real Time PCR and dPCR is the Real Time standard dependence. This create difficulties in the choice of standards, because it is necessary to verify the performance of the standards with respect to the samples analysed (based on the present results, it is not clear if it is possible to use a unique type of standard, for example constituted only of muscle, to detect target in products with different composition); application of dPCR could avoid using standards in the detection phase.

Copy number determination can be used to calculate target weight percentage in food product; if the method can be able to detect the same copy number, correspondent to a specific weight percentage in food of different composition, we could identify target weight percentage only by copy number measure. Improving dPCR detection in different composed food could allow to reach this aim; the correspondence between copy number and different weight percentages should be initially evaluated along a known concentrations range and in different types of products, to determine also the measure precision. This could be an alternative to the use of housekeeping gene, easier to apply in products based only on meat than in products containing also vegetables. However, it is important to underline that, in different species, copy number associated to weight percentage can change, maybe due to different genome size and consequently different cell number in a specific weight percentage; so, it becomes important to consider this variable when final target weight percentages calculation is performed. From data obtained in the present study, for the same weight percentage, avian genome copies are in average more than mammalian copies. The possibility to calculate, a “correction factor” to adjust weight percentage based on copy number value could allow to obtain directly the correct weight value, as an alternative to numerous experiments to associate copy number and weight percentage at least for one species group. The correction factor value, obtained from the ratio between avian and mammalian species, seems to be constant along the different percentages; on the other hand, the confidence intervals change as the concentrations vary, increasing towards extreme values, making impossible to use the correction factor for the previously described purpose. Confidence intervals could be reduced by increasing the sample size.

Finally, the analysis performed on meat-based commercial foods suggests the concrete possibility to apply dPCR to verify labels declaration. The system based on housekeeping gene seems efficient in products with only-meat ingredients; probably it could be difficult to apply that approach in case of multi-ingredients products (for example with meat and vegetables mixed).

## Methods

### Sample preparation and DNA extraction

Homogenized baby food purchased at the supermarket was selected for species identification analysis. Chicken baby food (Plasmon Dietetici Alimentari s.r.l., Milan, Italy) was selected as target of interest to be spiked at different levels in other seven baby food matrices. The label product reported: cooking water, chicken meat 30%, cornstarch, rice flour 2%, concentrated lemon juice). The other baby foods selected to produce chicken mixes had these labels:

1. Beef (Mellin Spa, Milan, Italy): cooking water, beef meat (30%), cornstarch, rice starch, rice flour (2.5%), sunflower oil, concentrated lemon juice.
2. Cheese (Hipp Italia s.r.l., Lainate, Italy): cooking water, cheeses from biological agriculture (43%: fresh quark cheese 30%, cheddar 8%, Parmigiano Reggiano 5%), rice starch, sodium citrate, gluten free
3. Veal meat (Alce Nero SpA, Bologna, Italy): cooking water, veal meat (40%), rice starch, lemon juice, gluten free from organic farming.
4. Mixed fruits (Alce Nero SpA, Bologna, Italy): fruit purees (93%: apple, pear, apricot), concentrated pear juice, ascorbic acid as antioxidant, gluten free from organic farming.
5. Mixed vegetables (Alce Nero SpA, Bologna, Italy): mixed vegetables in variable proportion (77%: carrots, potatoes, zucchini), cooking water, gluten free from organic farming.
6. Organic soy mayonnaise (Biobontà, Rivoli, Italy): sunflower oil, water, soy drink 8% (water, soy seeds dehulled 8%, Lithothamnium calcareum algae), apple vinegar, lemon juice, sugar cane, sea salt, mustard, thickeners (xanthan rubber and carob seed flour), gluten free from organic farming. It can contain eggs, nuts and fish traces
7. Yogurt (Granarolo, Bologna, Italy): whole milk, whole yogurt with live lactic acid bacteria Streptococcus thermophilus and lactobacillus bulgaricus).

All of these products were spiked with chicken baby food (30% starting meat) to obtain 1.5% and 15% (w/w) final concentrations of chicken meat. DNA extraction was performed on 200 mg of mixture with the Wizard® Genomic DNA Purification kit (Promega), according to the manufacturer’s instructions. Nine independent DNA extractions were done from each of spiked baby food.

For the correlation studies with dPCR, muscle samples of the main domestic species (avian and mammalian) were selected (chicken, turkey, horse, bovine and swine). DNA extraction was performed on 200 mg of muscle with the Wizard® Genomic DNA Purification kit (Promega) and diluted with deionized water to obtain 1:2, 1:4, 1:10, and 1:100 concentrations. From four to six different extractions were done for each species for each dilution.

### Quantification of extracted DNA content

The extracted DNA was quantified by Nanoquant Infinite M200 (Tecan), and all homogenized extracts were diluted to reach the same DNA concentration (10 ng/µL).

### Real time PCR assay

Amplification by Real Time PCR was performed by a CFX96™ Real Time PCR (BioRad) detection system. Chicken primers and probe were designed within a housekeeping gene^20–23^ and are reported in Table 5.

**Table 5:** Sequences of oligonucleotides used for chicken target in real time PCR and digital PCR, and for correlation studies.

Species	Denomination	Sequence 5′–3′
Chicken	Gallus-TGFB3-129bp-F	GGCTGCAAGTCACCGTGGTA
	Gallus-TGFB3-129bp-R	CCGCTAGCCAGAAGCTCAGC
	Gallus-TGFB3-129bp-P	FAM-CAGGAGCCACGTGAGCAGCACAG-MGB
Bovine	Bov_F2_digital For	CCTGTCTGCTGAGACGCCG
	Bov_F2_digital Rev	GTGGTAGAGTTGATTCTGGAATAGAAAGCAT
	Bov_F2 digital Probe	FAM—CCCCGCCACCCGCAGTGTCT—MGB
Horse	Eq_F2_digital For	GCCAGCAGGCTGAGAACG
	Eq_F2_digital Rev	GTGGTGCAGTTGATTCTGGAATAGGAAATTT
	Eq_F2 digital Probe	FAM—CCATGCCTCGCCCACCCTCA—MGB
Pig	Sus_F2_digital For	CTGCCAGCGGGCTGGGAATA
	Sus_F2_digital Rev	GGAGTTGACTCTGGAATAAGAAATTG
	Sus_F2 digital Probe	FAM—CGCCCCCGCCCCCAGGGTCT—MGB
Turkey	Turk_digital For	TGTATTTCAGTAGCACTGCTTATGACTACT
	Turk_digital Rev	TTTATTAATGCTGGAAGAATTTCCAA
	Turk digital Probe	FAM—TTATGGAGCATCGCTATCACCAGAAAA—MGB

Reactions were performed in a final volume of 20 µl composed by 1X qPCR Master mix (Promega), 0.3 µM of each primer, 0.25 µM probe, and 10 ng/µL of DNA. The thermal profile was 15′ at 95 °C, followed by 40 cycles of 30″ at 94 °C and 1′ at 60 °C. Each sample was amplified in three replicates. Three runs with three replicates each were performed to verify differences between contaminations (1.5 vs 15%) and intra-concentration variability; 15% spiked samples analysis was conducted to focus on a concentration more related to fraud than to trace contamination.

### Real time PCR standard curves

Two different types of standard curves were constructed, based respectively on two standard samples: chicken muscle (100% meat) and chicken baby food (30% meat). Both standards were extracted and the DNA concentration of muscle was measured by spectrophotometry; nanograms/µL DNA value of 30% chicken meat was obtained theoretically from chicken muscle DNA. The DNAs were diluted to obtain six curve points (log dilutions 10^–1^ to 10^–6^). In this way, the DNA content of each curve point was extrapolated from the Ct value of the dilutions. DNA concentrations (ng/µL) were converted to base 10 logarithms and fitted to the Ct values from Real Time PCR. Linear regression was applied to the obtained logarithmic values from samples, subsequently transformed into DNA nanograms/µL by exponential function.

#### Study of the effect of inhibitors on different matrices by Real Time PCR

In each single run of chicken-spiked baby foods standard curves were amplified in three different replicates, and Ct values of the different tested product were converted in nanograms/µL DNA.

### Digital PCR assays

#### Study of the effect of inhibitors on different matrices by dPCR

Digital PCR was carried out by a QuantStudio™ 3D Digital System (Thermofisher). dPCR was performed on the same spiked baby food samples and data were compared to Real Time PCR results.

#### Correlation studies

Digital PCR was also used to verify the difference in genome copies number between mammalian and avian species. The primers and probe for chicken target were the same used in the Real Time PCR assay; in Table 5 are listed all the primers used for the detection of avian and mammalian species (bovine, swine and horse for mammalian, chicken and turkey for avian species). Dilutions allowed to obtain different target percentages (100%, 50%, 25%, 10%, 1%), and all undiluted and diluted samples were amplified by digital PCR. DNA quantities to be inserted into the reaction were selected to achieve non-saturating conditions, according to the manufacter’s instruction.

The reaction was performed in a total volume of 16 µl containing 8 µL of QuantStudio™ 3D Digital PCR Master Mix v2, 0,9 µM each primer and 0,25 µM of the probe, and 5,3 µL of DNA. The reaction protocol was 10′ at 96 °C, followed by 39 cycles of 2′ at 60 °C and 30″ at 98 °C, and finally 60 °C for 2′. Each sample was tested in three replicates. Three different amplification reactions were performed separately for each species.

### Field samples analysis with dPCR

Three types of meat products from supermarkets were analyzed to verify the compliance of label with the declared species. In particular were selected:

1. Hamburger (78% bovine and 8% swine declared)
2. Meatballs (60% chicken plus turkey, and 19% swine declared)
3. Chicken Wurst (46% chicken and 38% turkey declared)

For this verification a housekeeping gene approach was applied. Degenerate oligonucleotides (Primer MyR 5′-ATACCAGTCCCTGGGTTCAT-3′; Primer MyF 5′-TTGTGCARATCCTGAGACTCAT-3′; probe CCCATGAAAGACGGTACAAGRTATACTG VIC-MGB) were used to amplify the myostatin genes^24^; to detect the species-specific target, oligonucleotide reported in Table 5 were used. Amplification was conducted in a final volume of 20 µl composed of 1X qPCR Master mix (Promega), 0.3 µM of each primer and 0.25 µM probe. The thermal profile was 15′ at 95 °C, followed by 40 cycles of 30″ at 94 °C and 1′ at 60°C.

Each field sample, extracted in three replicates, was amplified only once. Based on nanograms/µL of DNA extracted and species CV values, genome copies/µL obtained from Digital PCR was multiplied by a specific factor (3 for myostatin and 2 for other species). Finally, the ratio between the target genome copies and myostatin copies was calculated to obtain species target percentages.

### Statistical analysis

Data obtained from homogenized samples (DNA nanograms/µL) were evaluated by Kruskal Wallis test (normality and variance homogeneity were verified respectively by Shapiro Wilk test and Levene or Bartlett test); for post hoc test to detail differences among the different foods Dunn test for multiple comparisons was applied. The tests were applied on Real Time data, considering the two different standard curves, and also on digital data (genomic copies/µL).

The DNA content of muscle samples were extrapolated to obtain a 10 ng/µL concentration. dPCR data from the different species muscles (genomic copies/µL) were grouped by avian and mammalian species. The average number of genome copies was estimated using the negative binomial distribution (overdispersion assumption checked) considering animal species (poultry and mammal) and the concentration as an independent variable. The ratio between poultry and mammals with the relative 95% confidence interval (95% CI) was therefore calculated considering different weight concentrations. Results were used to evaluate the presence of a constant difference between the two groups in order to used it as a conversion factor to determine the correct weight percentages in field samples. All statistical analysis was performed using R (version 3.4.3) (R Core Team, 2018)^25^.

## Conclusions

Digital PCR seems to be a promising system for the application in the field of fraud control; further assessments should be done to better verify the quantification ability and how to improve system accuracy in different types of food. For a future development of the system, quantification ranges, limits, and uncertainty of measurement should be defined.

### Supplementary Information

Supplementary Tables.

## Data Availability

The data presented in this study are available in the Supplementary material section.

## Abbreviations

w/w: Weight by weight
EURL-AP: European Union Reference Laboratory for Animal proteins
PCR: Polymerase chain reaction
Ct: Cycle threshold
dPCR: Digital PCR
gc: Genome copies
