# Supplementary material for: Mammalian and avian species quantification in homogenized foods: real time PCR and digital PCR as tools for label compliance controls
Source: Sci Rep. 2024 May 9;14:10668. doi: 10.1038/s41598-024-61009-2 (PMC11082228; doi:10.1038/s41598-024-61009-2)
Supplement: Supplementary file 1 — Supplementary Tables. [file 41598_2024_61009_MOESM1_ESM.docx]

Table S1: Cycle threshold (Ct) obtained by real time PCR associated to spiked homogenized products and products used as standards (30% chicken meat homogenized and chicken muscle)

| Chicken percentage | Ct | | |
| --- | --- | --- | --- |
|  | Run 1 | Run 2 | Run 3 |
| 30% Chicken meat | 29,11 | 28,45 | 28,24 |
|  | 29,03 | 28,77 | 28,48 |
|  | 29,53 | 28,82 | 28,87 |
|  | 29,11 | 26,15 | 26,31 |
|  | 29,03 | 26,03 | 26,04 |
|  | 29,53 | 25,83 | 26,08 |
| Chicken muscle | 26,67 | 26,15 | 26,31 |
|  | 27,53 | 26,03 | 26,04 |
|  | 25,28 | 25,83 | 26,08 |
|  | 30,15 | 29,78 | 29,44 |
|  | 30,52 | 29,98 | 29,7 |
|  | 30,11 | 29,27 | 29,13 |
| 15% chicken in veal meat | 30,15 | 29,78 | 29,44 |
|  | 30,52 | 29,98 | 29,7 |
|  | 30,11 | 29,27 | 29,13 |
|  | 29,76 | 29,18 | 29,24 |
|  | 30,1 | 29,75 | 29,67 |
|  | 29,49 | 29,04 | 28,97 |
| 15% chicken in cheese | 29,76 | 29,18 | 29,24 |
|  | 30,1 | 29,75 | 29,67 |
|  | 29,49 | 29,04 | 28,97 |
|  | 30,19 | 29,66 | 29,41 |
|  | 30,65 | 30,11 | 29,89 |
|  | 29,99 | 29,4 | 29,34 |
| 15% chicken in soy mayonnaise | 30,19 | 29,66 | 29,41 |
|  | 30,65 | 30,11 | 29,89 |
|  | 29,99 | 29,4 | 29,34 |
|  | 30,09 | 29,65 | 29,4 |
|  | 29,06 | 28,71 | 28,45 |
|  | 29,24 | 28,79 | 28,53 |
| 15% chicken in yogurt | 30,09 | 29,65 | 29,4 |
|  | 29,06 | 28,71 | 28,45 |
|  | 29,24 | 28,79 | 28,53 |
|  | 30,51 | 30,21 | 30,09 |
|  | 30,43 | 29,95 | 29,94 |
|  | 30,33 | 29,8 | 29,6 |
| 15% chicken in mixed vegetables | 30,51 | 30,21 | 30,09 |
|  | 30,43 | 29,95 | 29,94 |
|  | 30,33 | 29,8 | 29,6 |
|  | 29,65 | 29,2 | 29,22 |
|  | 29,91 | 29,59 | 29,3 |
|  | 29,76 | 29,6 | 29,27 |
| 15% chicken in mixed fruits | 29,65 | 29,2 | 29,22 |
|  | 29,91 | 29,59 | 29,3 |
|  | 29,76 | 29,6 | 29,27 |
|  | 31,89 | 31,66 | 31,28 |
|  | 31,41 | 30,84 | 30,88 |
|  | 31,11 | 30,44 | 30,3 |
| 15% chicken in beef | 31,89 | 31,66 | 31,28 |
|  | 31,41 | 30,84 | 30,88 |
|  | 31,11 | 30,44 | 30,3 |
|  | 29,11 | 28,45 | 28,24 |
|  | 29,03 | 28,77 | 28,48 |
|  | 29,53 | 28,82 | 28,87 |

Table S2: Cycle threshold (Ct) obtained by real time PCR associated to spiked homogenized products and products used as standards

| Chicken percentage | Ct |
| --- | --- |
|  | Run 1 |
| 1,5% chicken in veal meat | 31,9 |
|  | 31,8 |
|  | 31,1 |
| 1,5% chicken in cheese | 30,8 |
|  | 30,7 |
|  | 30,2 |
| 1,5% chicken in soy mayonnaise | 33,7 |
|  | 33,6 |
|  | 33,5 |
| 1,5% chicken in yogurt | 31,8 |
|  | 32,3 |
|  | 31,9 |
| 1,5% chicken in mixed vegetables | 33,7 |
|  | 33,6 |
|  | 33,5 |
| 1,5% chicken in mixed fruits | 32,8 |
|  | 32,7 |
|  | 32,2 |
| 1,5% chicken in beef | 32,8 |
|  | 32,5 |
|  | 33,1 |

Table S3: Chicken DNA nanograms/µL, log DNA nanograms and Ct values to construct two types of linear standard curves and obtain equations in real time PCRs

| Chicken muscle | | Ct values | | |
| --- | --- | --- | --- | --- |
| Ng DNA | Log | Run 1 | Run 2 | Run 3 |
| 175000 | 5,243038 | 13,3 | 12,8 | 12,94 |
| 17500 | 4,243038 | 16,6 | 16,1 | 16,24 |
| 1750 | 3,243038 | 19,9 | 19,4 | 19,54 |
| 175 | 2,243038 | 23,2 | 22,7 | 22,84 |
| 17,5 | 1,243038 | 26,5 | 26 | 26,14 |
| 30% chicken meat | | Ct values | | |
| Ng DNA | Log | Run 1 | Run 2 | Run 3 |
| 52500 | 4,72E+00 | 16 | 15,48 | 15,33 |
| 5250 | 3,72E+00 | 19,3 | 18,78 | 18,63 |
| 525 | 2,72E+00 | 22,6 | 22,08 | 21,93 |
| 52,5 | 1,72E+00 | 25,9 | 25,38 | 25,23 |
| 5,25 | 7,20E-01 | 29,2 | 28,68 | 28,53 |
| 0,525 | -2,80E-01 | 32,5 | 31,98 | 31,83 |

Table S4: DNA nanograms/µL quantity obtained from the two type of standard curves, in three different real time PCR runs

|  | Chicken muscle curve | | | 30% chicken meat curve | | |
| --- | --- | --- | --- | --- | --- | --- |
|  | Run 1 | Run 2 | Run 3 | Run 1 | Run 2 | Run 3 |
| 15% chicken in veal meat | 1,37 | 1,25 | 1,75 | 2,71 | 2,44 | 2,78 |
|  | 1,06 | 1,09 | 1,46 | 2,09 | 2,12 | 2,32 |
|  | 1,41 | 1,79 | 2,17 | 2,78 | 3,48 | 3,46 |
| 15% chicken in cheese | 1,8 | 1,9 | 2,01 | 3,55 | 3,7 | 3,2 |
|  | 1,42 | 1,28 | 1,49 | 2,8 | 2,49 | 2,37 |
|  | 2,17 | 2,1 | 2,43 | 4,29 | 4,09 | 3,86 |
| 15% chicken in soy mayonnaise | 1,33 | 1,36 | 1,79 | 2,63 | 2,65 | 2,84 |
|  | 0,97 | 0,99 | 1,28 | 1,91 | 1,94 | 2,03 |
|  | 1,53 | 1,63 | 1,88 | 3,03 | 3,18 | 2,98 |
| 15% chicken in yogurt | 1,43 | 1,37 | 1,8 | 2,82 | 2,67 | 2,86 |
|  | 2,93 | 2,64 | 3,49 | 5,79 | 5,14 | 5,55 |
|  | 2,59 | 2,5 | 3,3 | 5,11 | 4,86 | 5,25 |
| 15% chicken in mixed vegetables | 1,07 | 0,93 | 1,11 | 2,11 | 1,81 | 1,77 |
|  | 1,13 | 1,11 | 1,23 | 2,23 | 2,16 | 1,96 |
|  | 1,21 | 1,23 | 1,57 | 2,39 | 2,4 | 2,49 |
| 15% chicken in mixed fruits | 1,94 | 1,88 | 2,04 | 3,84 | 3,65 | 3,24 |
|  | 1,62 | 1,43 | 1,93 | 3,2 | 2,78 | 3,07 |
|  | 1,8 | 1,42 | 1,97 | 3,55 | 2,76 | 3,13 |
| 15% chicken in beef | 0,41 | 0,34 | 0,48 | 0,8 | 0,66 | 0,77 |
|  | 0,57 | 0,6 | 0,64 | 1,12 | 1,16 | 1,02 |
|  | 0,7 | 0,79 | 0,96 | 1,39 | 1,54 | 1,53 |

Table S5: Products spiked with 15% of chicken target: Genome copies values obtained from digital PCR, in three different runs

| Spiked products | Genome copies/µL | | |
| --- | --- | --- | --- |
|  | Run 1 | Run 2 | Run 3 |
| 15% chicken in veal meat | 124,9 | 135,6 | 123,66 |
|  | 98,474 | 108,59 | 114,06 |
|  | 152,5 | 168,61 | 169,74 |
| 15% chicken in cheese | 139,57 | 188,54 | 188,75 |
|  | 198,51 | 145,31 | 158 |
|  | 178,33 | 204,95 | 208,57 |
| 15% chicken in soy mayonnaise | 145,2 | 152,13 | 138,5 |
|  | 115,72 | 118,31 | 118,35 |
|  | 180,47 | 199,79 | 194,69 |
| 15% chicken in yogurt | 143,43 | 138,69 | 141,85 |
|  | 315,9 | 323,1 | 275,8 |
|  | 246,21 | 252 | 246,16 |
| 15% chicken in mixed vegetables | 268,07 | 93,443 | 279,19 |
|  | 308,68 | 112,23 | 347,22 |
|  | 349,3 | 116,34 | 117,75 |
| 15% chicken in mixed fruits | 550,65 | 548,28 | 163,09 |
|  | 446,66 | 429 | 147,66 |
|  | 447,25 | 408,44 | 138,83 |
| 15% chicken in beef | 134,16 | 124,51 | 135,15 |
|  | 186,23 | 163,54 | 166,9 |
|  | 244,79 | 218,91 | 145,43 |

Table S6: Valid digital calls, based on 0,5 Quality Threshold (QT) respect to partition number in digital PCR. Products spiked with 15% of chicken target: genome copies values obtained from digital PCR, in three different runs

| Spiked products | Run 1 | | Run 2 | | Run 3 | |
| --- | --- | --- | --- | --- | --- | --- |
|  | Digital calls qualified  by QT | Partition number | Digital calls qualified  by QT | Partition number | Digital calls qualified  by QT | Partition number |
| 15% chicken in veal meat | 16701 | 18052 | 17326 | 18471 | 17347 | 18272 |
|  | 16543 | 17979 | 17998 | 18682 | 18077 | 18957 |
|  | 16816 | 17917 | 16231 | 17897 | 18006 | 18732 |
| 15% chicken in cheese | 15805 | 17349 | 17311 | 18634 | 15201 | 18279 |
|  | 16580 | 17629 | 17263 | 18305 | 17514 | 18517 |
|  | 17754 | 18781 | 16569 | 18534 | 16152 | 18903 |
| 15% chicken in soy mayonnaise | 16451 | 17615 | 16953 | 18013 | 17407 | 18451 |
|  | 16526 | 17656 | 17371 | 18515 | 16290 | 18320 |
|  | 16398 | 17600 | 18203 | 18987 | 17509 | 18366 |
| 15% chicken in yogurt | 17053 | 18053 | 16030 | 17218 | 18373 | 19119 |
|  | 17621 | 18864 | 16820 | 18114 | 17988 | 18679 |
|  | 16260 | 17565 | 16835 | 18091 | 18168 | 18832 |
| 15% chicken in mixed vegetables | 16944 | 17661 | 16904 | 17797 | 17532 | 18212 |
|  | 17469 | 18231 | 16957 | 17540 | 16523 | 17303 |
|  | 16620 | 17470 | 16763 | 17518 | 17221 | 18001 |
| 15% chicken in mixed fruits | 17241 | 17900 | 18232 | 19172 | 18346 | 19242 |
|  | 16807 | 17424 | 18106 | 18784 | 13022 | 13682 |
|  | 16010 | 17099 | 17681 | 18658 | 18165 | 18895 |
| 15% chicken in beef | 17504 | 18157 | 17528 | 18287 | 15404 | 16029 |
|  | 16147 | 17519 | 17090 | 18007 | 18002 | 18840 |
|  | 15907 | 16914 | 17043 | 18460 | 13293 | 14185 |

Table S7: Genome copies values obtained from digital PCR; muscles samples of different weight percentage; three mammalian species and two avian species

| Species | Weight percentage | Genome copies values | Species | Weight percentage | Genome copies values | Species | Weight percentage | Genome copies values |
| --- | --- | --- | --- | --- | --- | --- | --- | --- |
| bovine | 100 | 2838,5 | swine | 100 | 4303,2 | horse | 100 | 3146 |
| bovine | 100 | 2940,0 | swine | 100 | 4717,9 | horse | 100 | 2949 |
| bovine | 100 | 2279,5 | swine | 100 | 4684,4 | horse | 100 | 2756.2 |
| bovine | 100 | 1587,7 | swine | 100 | 4950,7 | horse | 50 | 1382.9 |
| bovine | 50 | 941,4 | swine | 50 | 2198,6 | horse | 50 | 1249.5 |
| bovine | 50 | 939,0 | swine | 50 | 2327,8 | horse | 50 | 1292.3 |
| bovine | 50 | 938,2 | swine | 50 | 2174,4 | horse | 50 | 1205.6 |
| bovine | 50 | 1035,2 | swine | 50 | 1880,6 | horse | 25 | 627.24 |
| bovine | 25 | 398,0 | swine | 25 | 1065,5 | horse | 25 | 586.76 |
| bovine | 25 | 415,7 | swine | 25 | 1133,3 | horse | 25 | 637.1 |
| bovine | 25 | 401,6 | swine | 25 | 1036,8 | horse | 25 | 703.93 |
| bovine | 25 | 402,8 | swine | 25 | 1053,7 | horse | 10 | 260.4 |
| bovine | 10 | 181,8 | swine | 10 | 370,7 | horse | 10 | 263.34 |
| bovine | 10 | 166,7 | swine | 10 | 339,5 | horse | 10 | 292.72 |
| bovine | 10 | 215,2 | swine | 10 | 370,4 | horse | 10 | 23.966 |
| bovine | 10 | 213,8 | swine | 1 | 41,8 | horse | 1 | 24.978 |
| bovine | 1 | 20,0 | swine | 1 | 41,0 | horse | 1 | 27.623 |
| bovine | 1 | 17,8 | swine | 1 | 44,4 | horse | 1 | 2.506 |
| bovine | 1 | 25,2 | swine | / | / | horse | 1 | 24.974 |
| bovine | 1 | 21,6 | swine | / | / | / | / | / |
| chicken | 100 | 9282,7 | turkey | 100 | 9669,8 | / | / | / |
| chicken | 100 | 10776,0 | turkey | 100 | 9224,1 | / | / | / |
| chicken | 100 | 9726,9 | turkey | 100 | 8014,9 | / | / | / |
| chicken | 100 | 11650,0 | turkey | 100 | 9065,1 | / | / | / |
| chicken | 50 | 6245,0 | turkey | 50 | 3781,9 | / | / | / |
| chicken | 50 | 5271,0 | turkey | 50 | 2108,9 | / | / | / |
| chicken | 50 | 5151,3 | turkey | 50 | 3912,4 | / | / | / |
| chicken | 50 | 5093,6 | turkey | 50 | 3810,7 | / | / | / |
| chicken | 50 | 3816.9 | turkey | 50 | 3767.7 | / | / | / |
| chicken | 50 | 4320.8 | turkey | 50 | 3977.9 | / | / | / |
| chicken | 25 | 2604,6 | turkey | 25 | 2271,0 | / | / | / |
| chicken | 25 | 2175,8 | turkey | 25 | 2399,9 | / | / | / |
| chicken | 25 | 2776,2 | turkey | 25 | 2329,0 | / | / | / |
| chicken | 25 | 2875,6 | turkey | 25 | 1992,2 | / | / | / |
| chicken | 25 | 2036.2 | turkey | 25 | 2032.1 | / | / | / |
| chicken | 25 | 2051.4 | turkey | 25 | 2118.1 | / | / | / |
| chicken | 10 | 1540,1 | turkey | 10 | 739,0 | / | / | / |
| chicken | 10 | 1679,7 | turkey | 10 | 850,9 | / | / | / |
| chicken | 10 | 1627,8 | turkey | 10 | 777,3 | / | / | / |
| chicken | 10 | 788.92 | turkey | 10 | 840,3 | / | / | / |
| chicken | 10 | 701.57 | turkey | 10 | 808.78 | / | / | / |
| chicken | 1 | 161,7 | turkey | 10 | 690.14 | / | / | / |
| chicken | 1 | 142,2 | turkey | 1 | 69,0 | / | / | / |
| chicken | 1 | 148,7 | turkey | 1 | 76,5 | / | / | / |
| chicken | 1 | 118,2 | turkey | 1 | 72,3 | / | / | / |
| chicken | 1 | 75.503 | turkey | 1 | 82,4 | / | / | / |
| chicken | 1 | 74.067 | turkey | 1 | 70.711 | / | / | / |

Table S8: Valid digital calls, based on 0,5 Quality Threshold (QT) respect to partition number in digital PCR; muscles samples of different weight percentage; three mammalian species and two avian species

| Weight  % bovine | Digital calls qualified by QT | Partition number | Weight  % swine | Digital calls qualified by QT | Partition number | Weight % horse | Digital calls qualified by QT | Partition number |
| --- | --- | --- | --- | --- | --- | --- | --- | --- |
| 100 | 16890 | 17651 | 100 | 17173 | 18043 | 100 | 17591 | 18535 |
| 100 | 18538 | 19401 | 100 | 17126 | 18076 | 100 | 18717 | 19404 |
| 100 | 17889 | 18651 | 100 | 15811 | 17274 | 100 | 17780 | 18530 |
| 100 | 15395 | 15958 | 100 | 13190 | 17800 | 50 | 15458 | 16023 |
| 50 | 16284 | 17609 | 50 | 14142 | 17997 | 50 | 18728 | 19409 |
| 50 | 16086 | 16977 | 50 | 14539 | 17853 | 50 | 18828 | 19530 |
| 50 | 15760 | 17349 | 50 | 11093 | 18425 | 50 | 16744 | 19183 |
| 50 | 14973 | 16202 | 50 | 17032 | 18018 | 25 | 17768 | 18974 |
| 25 | 14658 | 17382 | 25 | 16780 | 17773 | 25 | 17844 | 18646 |
| 25 | 16733 | 17594 | 25 | 16787 | 17941 | 25 | 16685 | 19071 |
| 25 | 16511 | 17523 | 25 | 17046 | 18068 | 25 | 15035 | 18728 |
| 25 | 16939 | 18047 | 25 | 15998 | 18067 | 10 | 17975 | 19094 |
| 10 | 14091 | 16876 | 10 | 13199 | 17449 | 10 | 18523 | 19182 |
| 10 | 16176 | 17565 | 10 | 14901 | 18004 | 10 | 17138 | 18280 |
| 10 | 12244 | 13552 | 10 | 12728 | 17933 | 10 | 18151 | 18724 |
| 10 | 16235 | 16896 | 1 | 17124 | 17921 | 1 | 17922 | 18915 |
| 1 | 14144 | 15697 | 1 | 16167 | 17695 | 1 | 18088 | 18754 |
| 1 | 16986 | 17532 | 1 | 15745 | 17931 | 1 | 17639 | 18164 |
| 1 | 15497 | 16260 | / | / | / | 1 | 18549 | 19142 |
| 1 | 17518 | 18176 | / | / | / | / | / | / |
| Weight  % chicken | Digital calls qualified by QT | Partition number | Weight  % turkey | Digital calls qualified by QT | Partition number | / | / | / |
| 100 | 17128 | 17666 | 100 | 17267 | 17974 | / | / | / |
| 100 | 16162 | 17320 | 100 | 16202 | 17370 | / | / | / |
| 100 | 15539 | 16867 | 100 | 17674 | 18254 | / | / | / |
| 100 | 16355 | 17216 | 100 | 15503 | 17577 | / | / | / |
| 50 | 16224 | 17356 | 50 | 17060 | 17947 | / | / | / |
| 50 | 16963 | 17666 | 50 | 13848 | 16422 | / | / | / |
| 50 | 16162 | 16750 | 50 | 16286 | 17621 | / | / | / |
| 50 | 16195 | 16972 | 50 | 15729 | 16639 | / | / | / |
| 50 | 17257 | 18379 | 50 | 16882 | 18204 | / | / | / |
| 50 | 16671 | 17474 | 50 | 17762 | 18952 | / | / | / |
| 25 | 17195 | 17740 | 25 | 9433 | 16684 | / | / | / |
| 25 | 17183 | 18135 | 25 | 7838 | 14325 | / | / | / |
| 25 | 16406 | 17150 | 25 | 7984 | 14327 | / | / | / |
| 25 | 17524 | 18573 | 25 | 16807 | 18572 | / | / | / |
| 25 | 16983 | 17812 | 25 | 15139 | 18443 | / | / | / |
| 25 | 16281 | 17745 | 25 | 7086 | 16074 | / | / | / |
| 10 | 16196 | 17723 | 10 | 14013 | 16956 | / | / | / |
| 10 | 16599 | 17737 | 10 | 13286 | 16456 | / | / | / |
| 10 | 15528 | 17038 | 10 | 13596 | 16595 | / | / | / |
| 10 | 16916 | 17795 | 10 | 13375 | 16585 | / | / | / |
| 10 | 17266 | 18375 | 10 | 15740 | 18757 | / | / | / |
| 1 | 16034 | 17170 | 10 | 14919 | 17585 | / | / | / |
| 1 | 16766 | 17721 | 1 | 16675 | 17608 | / | / | / |
| 1 | 16612 | 17351 | 1 | 16110 | 16718 | / | / | / |
| 1 | 15911 | 17104 | 1 | 16908 | 17843 | / | / | / |
| 1 | 15859 | 17355 | 1 | 16469 | 17299 | / | / | / |
| 1 | 15446 | 17009 | 1 | 17088 | 17467 | / | / | / |

Table S9: Genome copies/µL values obtained from digital PCR from different commercial products (hamburger, meatballs and wurstel of different species) and myostatin genome copies/µL values

| Products | Myostatin | Bovine | Swine | Chicken | Turkey |
| --- | --- | --- | --- | --- | --- |
| meatballs | 27894 | 19474 | 1942 | / | / |
| meatballs | 31182 | 23172 | 1846 | / | / |
| meatballs | 28806 | 22114 | 1869 | / | / |
| hamburger | 40011 | / | 2975 | 174 | 35052 |
| hamburger | 38142 | / | 3011 | 158 | 20318 |
| hamburger | 30831 | / | 3106 | 214 | 31562 |
| Wurst | 73419 | / | / | 32661 | 12410 |
| Wurst | 58518 | / | / | 32364 | 17522 |
| Wurst | 52611 | / | / | 34104 | 14576 |

Table S10: Valid digital calls, based on 0,5 Quality Threshold (QT) respect to partition number in digital PCR. Genome copies/µL values obtained from digital PCR from different commercial products (hamburger, meatballs and wurstel of different species) and myostatin genome copies/µL values

| Products | Myostatin | | Bovine | | Swine | |
| --- | --- | --- | --- | --- | --- | --- |
|  | Digital calls qualified by QT | Partition number | Digital calls qualified by QT | Partition number | Digital calls qualified by QT | Partition number |
| meatballs | 13093 | 14194 | / | / | 7913 | 16522 |
| meatballs | 16447 | 17856 | / | / | 8008 | 16915 |
| meatballs | 14439 | 16627 | / | / | 8046 | 17351 |
| hamburger | 14661 | 16745 | 15353 | 16735 | 16226 | 17420 |
| hamburger | 11601 | 13761 | 16582 | 17664 | 16943 | 17575 |
| hamburger | 14424 | 16308 | 16839 | 17577 | 16319 | 17613 |
| Wurst | 13268 | 14466 | 13268 | 14466 | / | / |
| Wurst | 12138 | 14420 | 12138 | 14420 | / | / |
| Wurst | 15414 | 17281 | 15414 | 17281 | / | / |
|  | Chicken | | Turkey | |  |  |
|  | Digital calls qualified by QT | Partition number | Digital calls qualified by QT | Partition number |  |  |
| meatballs | 14125 | 16157 | 15770 | 16725 | / | / |
| meatballs | 14840 | 16110 | 15852 | 16834 | / | / |
| meatballs | 14789 | 17188 | 15376 | 16894 | / | / |
| hamburger | / | / | / | / | / | / |
| hamburger | / | / | / | / | / | / |
| hamburger | / | / | / | / | / | / |
| Wurst | 15798 | 16453 | 15108 | 16427 | / | / |
| Wurst | 15526 | 16438 | 15596 | 16609 | / | / |
| Wurst | 14271 | 15786 | 14429 | 16178 | / | / |
